# Pancreatolithiasis with an anatomic variant of Wirsung duct: a case report

**DOI:** 10.1093/gastro/goad065

**Published:** 2023-10-26

**Authors:** Satoshi Yamamoto, Kazuo Inui, Yoshiaki Katano, Senju Hashimoto, Hironao Miyoshi, Takashi Kobayashi, Yoshihiko Tachi

**Affiliations:** Department of Gastroenterology, Fujita Health University Bantane Hospital, Otoubashi, Nakagawa-ku, Nagoya, Aichi, Japan; Department of Gastroenterology, Yamashita Hospital, Nakamachi, Ichinomiya, Aichi, Japan; Department of Gastroenterology, Fujita Health University Bantane Hospital, Otoubashi, Nakagawa-ku, Nagoya, Aichi, Japan; Department of Gastroenterology, Fujita Health University Bantane Hospital, Otoubashi, Nakagawa-ku, Nagoya, Aichi, Japan; Board Chairman, Geriatric Health Services Facility Yuki, Mizuhomachi, Handa, Aichi, Japan; Department of Gastroenterology, Fujita Health University Bantane Hospital, Otoubashi, Nakagawa-ku, Nagoya, Aichi, Japan; Department of Gastroenterology, Fujita Health University Okazaki Medical Center, Harisakicho, Okazaki, Aichi, Japan

## Introduction

In contrast to familiar developmental anomalies, such as pancreatobiliary maljunction and pancreas divisum, a double orifice of the ampulla of Vater is a much more rare congenital malformation. We encountered a patient with pancreatolithiasis associated with anatomic variation of Wirsung duct showing a single major duodenal papilla with a double orifice and no minor papilla.

## Case report

A 69-year-old man complained of back pain during a follow-up examination after a gastric carcinoid had been endoscopically resected. Computed tomography and endoscopic ultrasonography demonstrated a 15-mm pseudocyst in the pancreatic head and a 9.5-mm pancreatic stone in front of the cyst.

**Figure 1.**
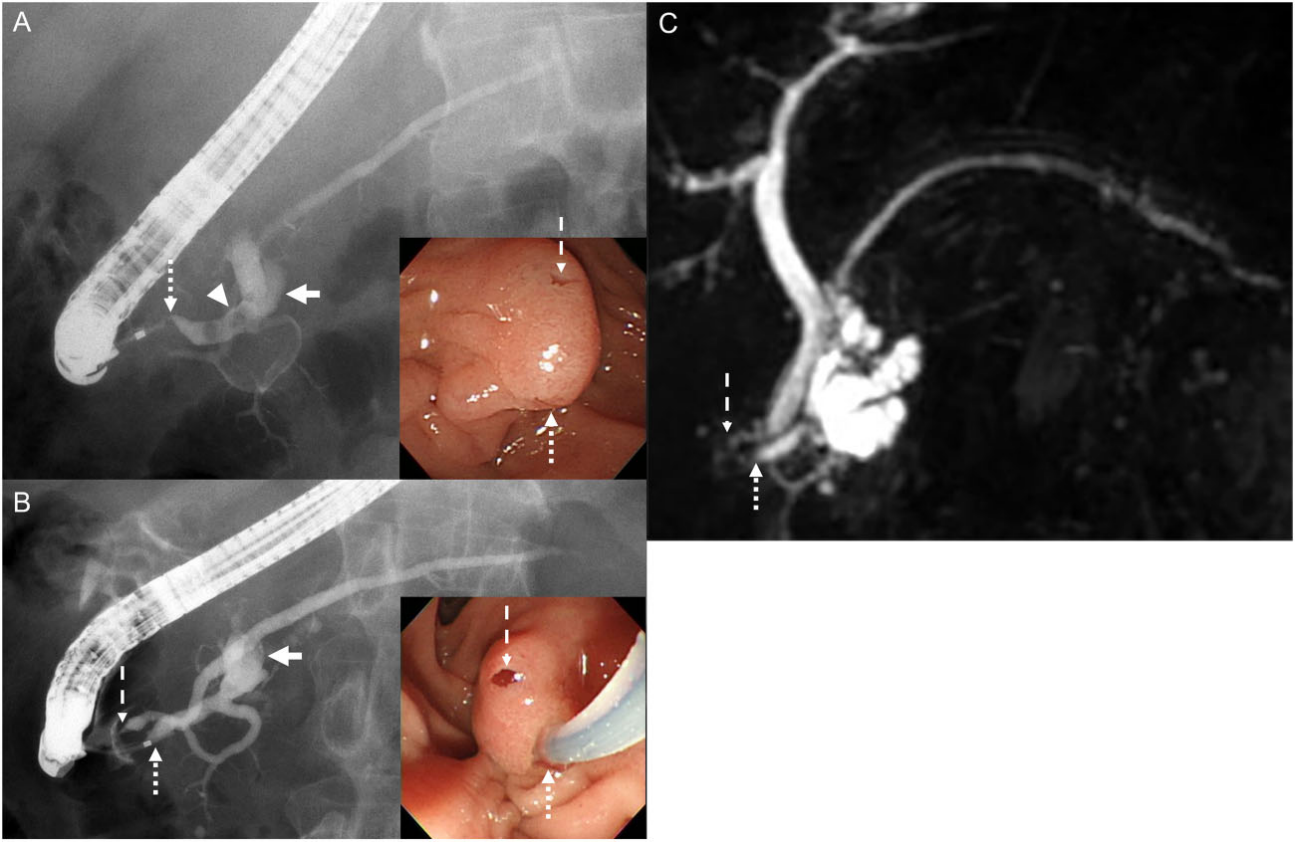
Endoscopic retrograde pancreatographic endoscopic findings and magnetic resonance cholangiopancreatography. (A) Endoscopic retrograde pancreatographic and endoscopic findings at our hospital prior to treatment of pancreatolithiasis. The stone (arrowhead) and the pseudocyst (arrow) in the main pancreatic duct are visualized by using endoscopic retrograde pancreatography via the main pancreatic duct (dotted line arrow). A fistula-like opening upon a longitudinal fold above the major duodenal papilla is evident endoscopically (dashed line arrow). (B) Endoscopic retrograde pancreatographic and endoscopic findings after extracorporeal shock wave lithotripsy. Pancreatography (dotted line arrow) shows the main pancreatic duct looping inferiorly toward the major duodenal papilla and giving rise to a branch with an orifice above the major duodenum papilla and the pseudocyst (arrow). Endoscopy demonstrates contrast material emerging from a fistula-like channel in the longitudinal fold above the major duodenal papilla (dashed line arrow). (C) Magnetic resonance cholangiopancreatography before treatment. The common bile duct communicated with the main pancreatic duct (dotted line arrow) and a fistula-like channel opened to the left side of the common bile duct (dashed line arrow).

Upon referral to our hospital for treatment, the patient noted mild back pain. Body temperature was 36.1°C and blood pressure was 132/83 mmHg. Endoscopic retrograde pancreatography showed a fistula-like channel leading to the longitudinal fold of the major duodenal papilla, as well as filling defects representing a stone in the main pancreatic duct. The pseudocyst was slightly proximal to the stone (Figure 1A). We diagnosed pancreatolithiasis with pseudocyst formation and delivered 3,100 pulses of extracorporeal shock wave lithotripsy as nonsurgical treatment. After treatment, the fragmentation of the stone caused mild acute pancreatitis, but pain soon abated with conservative treatment. However, computed tomography showed that a stone fragment had migrated to a position near the outlet of the main pancreatic duct.

After endoscopic extraction of the stone fragment, pancreatography showed the main pancreatic duct looping inferiorly toward the major duodenal papilla and giving off a branch leading to an orifice just above the papilla (Figure 1B). No minor duodenal papilla was evident; instead, contrast material emerged from a fistula-like channel in a longitudinal fold just above the major papilla. These findings represented an anatomic variant of Wirsung duct (Figure 1B). Computed tomography after the patient was discharged showed disappearance of the stone and the pseudocyst.

## Discussion

Formation of extrahepatic bile ducts occurs during a process of recanalization, when the common bile duct and pancreatic duct join at the major papilla and open into the duodenum; if they fail to join, they open separately into the duodenum [1].

A search for cases of double orifice reported from 1964 to 2023 disclosed 15 cases reported in 10 papers [1–10]. Simon *et al.* [2] reported that a double orifice was found in 3 of 1,600 (0.18%) cases in which endoscopic retrograde cholangiopancreatography was performed, representing a rarity. Describing relationships of the common bile duct and the pancreatic duct in that situation, Kothadia *et al.* [5] reported five types of double-orifice cases, all with connections of the main pancreatic duct and bile duct to orifices. All cases showed abnormalities of the common bile duct, and some of them had two bile duct orifices. However, none had two openings of the pancreatic duct. In our case, magnetic resonance cholangiopancreatography before treatment for pancreatolithiasis showed no anomaly of the common bile duct, but the common bile duct was found to communicate with the main pancreatic duct. The fistula was found to enter the duodenum to the left of the common bile duct (Figure 1C). Endoscopic observation located the fistula opening directly rostral to the main pancreatic duct opening (Figure 1A and B). Thus, two pancreatic duct openings emerged from a single major papilla—a configuration that we believe differs from double-orifice types reported in the past.

No accessory pancreatic duct was evident in our case. This raises the possibility that drainage of pancreatic juice through the fistula was poorer than that through the pancreatic duct in individuals with typical anatomy. Such a situation could have favored the development of pancreatolithiasis.

## Conclusion

A double orifice at the ampulla Vater is a rare congenital malformation. Our patient’s findings appear distinct from those described previously.

## Authors’ Contributions

S.Y.: Conceptualization; Data curation; Investigation; Resources; Writing—review & editing; Writing—original draft. K.I.: Conceptualization; Supervision; Project administration; Writing—review & editing. Y.K.: Project administration; Supervision. S.H.: Project administration; Writing—review & editing. H.M.: Data curation; Supervision. T.K.: Data curation; Supervision. Y.T.: Supervision. All authors read and approved the final manuscript. All authors have consented to the publication of this manuscript.
